# Tolerating cardiovascular health disparities is a failure of science and society

**DOI:** 10.1016/j.lanepe.2025.101445

**Published:** 2025-08-21

**Authors:** The Lancet Regional Health – Europe

Cardiovascular disease is responsible for more than 40% of all deaths in Europe. Decades of research and investment have achieved impressive declines in overall cardiovascular disease mortality, yet this progress hides a persistent injustice: the benefits of prevention, diagnosis, and treatment have not been shared equally across different strata of populations. Avoidable inequalities in cardiovascular health continue to claim lives, undermine health and wellbeing, and limit individuals’ opportunities to lead full, productive lives, exposing deep structural flaws in our health systems and research agendas.

To confront these inequities, *The Lancet Regional Health*—*Europe* has launched a Series on Inequalities and Disparities in Cardiovascular Health, as part of ongoing work towards a Commission. This Series brings together leading experts to examine the evidence on four groups that face persistent disparities in cardiovascular health: women, ethnic and racial minorities, older people, and people with mental health conditions. Each paper in this Series explores the unique and overlapping barriers these groups face—from bias and underrepresentation in research, to reduced access to health care and systemic failures in its delivery, to the compounding effects of social and economic disadvantage. Collectively, the Series provides a set of recommendations to address these disparities.

The inequalities are striking. Women have long been underdiagnosed, undertreated, and underrepresented in cardiovascular disease research. Although women generally develop ischaemic heart disease later than men, their mortality rates are paradoxically higher, even in high-income countries. For example, in 2019, women in Germany and Austria were about 25–30% more likely to die from ischaemic heart disease than men of the same age. Studies too often assume that the typical cardiovascular patient is male, and clinical guidelines and risk scores reflect this bias. Delays in diagnosis and treatment, particularly for acute myocardial infarction, are not merely common—they are lethal. The burden is compounded by psychosocial stressors, caregiving responsibilities, and socioeconomic disadvantage, all of which disproportionately affect women.

For people from ethnic minorities and Indigenous peoples, the legacy of social exclusion, discrimination, and structural racism is evident in the numbers. In one study from the UK, South Asian populations had a 70% higher risk of coronary heart disease than people identifying as European, even after adjusting for age and sex. Across Europe and North America, ethnic minorities and Indigenous populations experience persistent obstacles to prevention, diagnosis, and care—ranging from language and cultural barriers to financial and geographical exclusion.

Older people, meanwhile, are the fastest-growing demographic group in Europe and account for the vast majority of cardiovascular disease cases and deaths: more than 80% of cardiovascular deaths in women and over half in men occur in those aged 75 years and above. Yet clinical trials and guidelines have consistently excluded or underrepresented those older than 75 years, leaving clinicians to extrapolate from evidence that does not reflect the complexity of multimorbidity, frailty, and polypharmacy in later life. Older patients are more likely to encounter diagnostic uncertainty, social isolation, and financial hardship than younger patients, further exacerbating risk.

Perhaps the most neglected inequality is endured by people with mental health conditions. This group faces a 10–20-year reduction in life expectancy, largely due to preventable cardiovascular disease. The relationship between mental health and cardiovascular disease is bidirectional and mutually reinforcing, yet mental health disorders remain systematically overlooked in research, risk prediction, and routine care. People with mental health conditions are less likely to be screened or treated for cardiovascular risk factors and are more likely to face stigma and receive fragmented care.

A recurring theme throughout the Series is intersectionality—the recognition that the four groups addressed in the papers are not mutually exclusive. Many individuals belong to more than one group and therefore face overlapping challenges, which can amplify vulnerability and reduce access to appropriate care.

The Series makes clear what must be done. Research needs to reflect the true diversity of our populations, with inclusion of women, people from ethnic minorities, older adults, and people with mental health conditions in clinical trials. Health systems must move beyond one-size-fits-all models and embrace truly person-centred, culturally competent, and interdisciplinary care. Governments and funders must invest in collection of data on self-reported ethnicity and race, age, and mental health, in a safe and unstigmatising way, and use these data to target high-risk groups for prevention, screening, and treatment. Clinical education must confront bias and stigma, and patients and communities must be included as partners in research, policy, and care.

Tolerating cardiovascular health disparities is a failure of science and society. No one should be denied the benefits of advances in cardiovascular care because of their sex, ethnicity, age, or mental health. Allowing these disparities to persist in the face of clear evidence and effective solutions is costing lives. As this Series demonstrates, equity in cardiovascular health is not merely an aspiration but an urgent imperative.

